# CAPN15 is a non-proteasomal, ubiquitin-directed calpain protease that regulates cell adhesion by cleaving E-cadherin

**DOI:** 10.1016/j.jbc.2025.111034

**Published:** 2025-12-09

**Authors:** Aya Noguchi, Hikaru Tsuchiya, Hiroshi Shitara, Yasushi Saeki, Yasuko Ono, Shoji Hata

**Affiliations:** 1 Calpain Project, Department of Basic Medical Sciences, Tokyo Metropolitan Institute of Medical Science (TMiMS), Tokyo, Japan; 2 Department of Physiology, Juntendo University Graduate School of Medicine, Tokyo, Japan; 3 Autophagy Research Center, Juntendo University Graduate School of Medicine, Tokyo, Japan; 4 Protein Metabolism Project, Department of Basic Medical Sciences, Tokyo Metropolitan Institute of Medical Science (TMiMS), Tokyo, Japan; 5 Animal Research Division, Center for Basic Technology Research, Tokyo Metropolitan Institute of Medical Science (TMiMS), Tokyo, Japan; 6 Division of Protein Metabolism, Department of Basic Medical Sciences, The Institute of Medical Science, The University of Tokyo, Tokyo, Japan; 7 Research Administration Center, Saitama Medical University, Saitama, Japan

**Keywords:** calpain, ubiquitin, ubiquitin-directed protease, cadherin, cell adhesion

## Abstract

CAPN15, a member of the calpain protease family, contains a unique N-terminal region with five Zn^2+^-finger domains, including two ubiquitin-binding Npl4-type Zn^2+^-finger domains. However, the role of these domains in CAPN15 function remains unknown. In this study, we show that CAPN15 functions as a non-proteasomal, ubiquitin-directed protease, regulating cell surface E-cadherin. In cultured epithelial cells, *CAPN15* KO resulted in densely packed morphology accompanied by the accumulation of cell surface E-cadherin, suggesting an increased cell adhesion. This defect was rescued by expressing WT CAPN15, but not protease-inactive or ubiquitin-binding-deficient CAPN15. CAPN15 recognizes the ubiquitinated E-cadherin-catenin complex through its N-terminal Zn^2+^-finger region and cleaves E-cadherin near its transmembrane domain. Since the truncated form is directed for lysosomal degradation, CAPN15 stands as a negative regulator of E-cadherin. E-cadherin accumulation was also observed in the epithelial tissues of *Capn15* KO mice, further corroborating the *in vivo* relevance of CAPN15 to a mechanism controlling E-cadherin function. These findings reveal a previously unappreciated ubiquitin-dependent proteolytic pathway involving CAPN15 and provide important insight into the regulation of cell adhesion.

Calpains (CAPNs) are a family of intracellular Ca^2+^-dependent cysteine proteases with a highly conserved protease domain and they are present in a wide range of organisms (1, 2). In humans, the 15 calpain genes exhibit various expression patterns and molecular structures. Calpains cleave substrates in a limited manner to modulate their structures or functions, thereby regulating multiple biological processes including apoptosis, the cell cycle, neuronal remodeling, and myoblast fusion. Accordingly, the malfunction of calpain activity is associated with various pathological states, including developmental failure, neurodegenerative diseases, and muscular dystrophies (2, 3, 4, 5, 6).

CAPN15, also known as SOLH, is a mammalian homolog of *Drosophila* Small Optic Lobes (SOL), whose mutation causes neurodegeneration in the developing optic lobes (7, 8, 9). Recently, several *CAPN15* pathological variants have been identified in individuals with oculogastrointestinal neurodevelopmental syndrome (OGIN) (OMIM #619318), an autosomal recessive congenital disorder characterized by microphthalmia or anophthalmia with systemic anomalies such as hearing loss and developmental delay (10, 11, 12, 13). Similarly, *Capn15* KO mice exhibit growth retardation with eye hypoplasia, cataracts, and smaller brains (10, 14, 15), suggesting that the loss of CAPN15 function contributes to the pathogenesis of OGIN.

Calpains homologous to SOL are found in almost all metazoans and constitute the SOL subfamily. In the calpain family, SOL calpains have a unique N-terminal region containing multiple Zn^2+^-finger (ZF) domains and a C-terminal SOL-homologous domain, in addition to a conserved calpain protease domain (Fig. 1*A*). The ZF domain is a Zn^2+^-binding module comprising of approximately 30 amino acid residues, including four conserved cysteine residues that coordinate with a Zn^2+^ ion. Notably, some ZF domains match the consensus sequence of the Npl4-type ZF (NZF) domain, characterized by a Thr-(Phe or Tyr) motif (referred to as the TF motif). The TF motif, together with a hydrophobic residue located 10 residues apart, has been shown to contribute to ubiquitin-binding (Fig. 1*B*) (16, 17, 18). In fact, the ZF region of *Aplysia* SOL was bound to polyubiquitin *in vitro* (19), suggesting that interaction with ubiquitin is functionally important for SOL calpains in diverse animal species. Proteins containing ubiquitin-binding domains specifically recognize substrate-conjugated mono- or polyubiquitins, which determines the fate of the substrate and regulates specific cellular processes, such as protein degradation by the proteasome (20). Considering that CAPN15 is also an intracellular protease, it is tempting to speculate a role of ubiquitin in the process of substrate cleavage by CAPN15. However, the function of CAPN15 and its relevance to ubiquitin-binding have been left unexplored.

**Figure 1:**
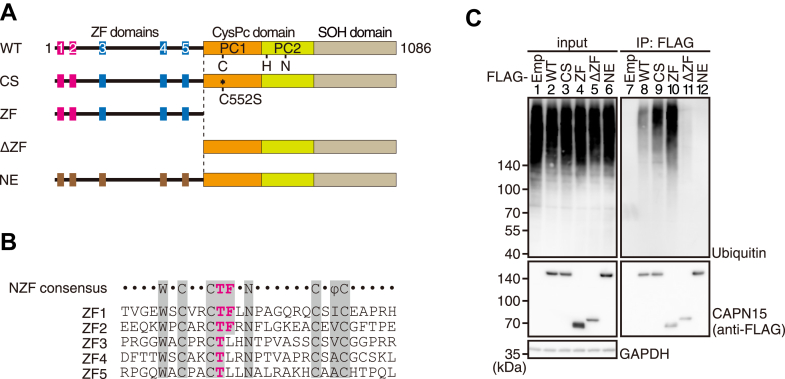
**The binding of ubiquitinated proteins to CAPN15 is dependent on the NZF domains.***A*, schematic of the domain structures of human CAPN15 (WT) and its mutants (CS, ZF, ΔZF, and NE). The calpain-type cysteine protease conserved (CysPc) domain is a cysteine protease domain composed of protease core subdomains one and 2 (PC1 and PC2), in which C552 (C), H717 (H), and N737 (N) form the catalytic triad. CS, a protease-inactive C552S mutant; NE, a mutant in which the TF motifs in ZF1 and ZF2 domains and the corresponding T-L dipeptides in ZF3-5 domains were substituted with NE. SOH, SOL homologous. *B*, alignment of ZF domains with the NZF consensus sequence, with residues identical to the consensus sequence shown with shading. The TF motif was labeled in magenta. Φ indicates hydrophobic residues. *C*, immunoprecipitation of ubiquitinated proteins with FLAG-CAPN15 constructs. They were transfected into HCT116 cells, and the cell lysates were subjected to immunoprecipitation with anti-FLAG antibodies followed by western blotting. Emp, empty vector. ZF, Zn^2+^-finger; NZF, Npl4-type ZF.

In this study, we found that, in epithelial cells, CAPN15 recognized the E-cadherin-catenin cell adhesion complex through the ZF region in a ubiquitination-dependent manner, and cleaved E-cadherin, leading to its downregulation. Conversely, loss of CAPN15 resulted in E-cadherin accumulation. We also observed that *CAPN15* KO cells did not exhibit the accumulation of ubiquitinated proteins, as is typically observed when the proteasome is inhibited, suggesting that ubiquitination-dependent substrate cleavage by CAPN15 does not overlap with the proteasomal protein degradation. Our findings demonstrated that CAPN15 is a ubiquitin-directed calpain protease that targets E-cadherin.

## Results

### CAPN15 binds to ubiquitinated proteins

CAPN15 contains two NZF domains (ZF1 and ZF2) and three RanBP2-type ZF domains (ZF3-5) in its N-terminal region (Fig. 1, *A* and *B*) (18). To examine whether CAPN15 binds to ubiquitinated proteins, immunoprecipitation was performed using an anti-FLAG antibody in HCT116 cells transfected with FLAG-tagged human CAPN15 constructs, as shown in Figure 1*A*. In this experiment, we used two different constructs in which ubiquitin interaction is expected to be abrogated: one lacks the ZF region (ΔZF), and the other carries substitution mutations that replace each of the TF motif and the corresponding TL dipeptide with NE in the 5 ZF domains (NE) (16). The result showed that ubiquitinated proteins were immunoprecipitated with FLAG-tagged WT CAPN15 (WT), FLAG-tagged protease-inactive CAPN15C552S (CS), and FLAG-tagged CAPN15 ZF region (ZF), but not with FLAG-tagged ΔZF or FLAG-tagged NE, indicating that CAPN15 binds to ubiquitinated proteins through the ZF domains (Fig. 1*C*; see Table S1 for abbreviations).

To analyze the ubiquitin-binding ability of the ZF domains, we performed a GST pull-down assay using unanchored K48- and K63-linked ubiquitin chains (Fig. S1). Polyubiquitins, but not mono- or di-ubiquitin, were preferentially pulled down using the GST-tagged ZF constructs (Fig. S1*A*). ZF1-3 and ZF1-4 showed binding abilities for K48- and K63-linked ubiquitin chains comparable to that of ZF1-5, suggesting that ZF4 and ZF5 are dispensable (Fig. S1, *B* and *D*). Furthermore, ZF1-2 exhibited a binding ability comparable to that of ZF1-3, while the binding abilities of ZF2 and ZF3 were faint compared to that of ZF1 (Fig. S1, *C* and *E*). These results indicated that CAPN15 prefers longer ubiquitin chains, with the binding ability mostly attributed to the ZF1 domain.

Notably, ubiquitinated proteins immunoprecipitated with FLAG-CS were found to be more abundant than those immunoprecipitated with FLAG-WT (Fig. 1*C*, lanes 8 and 9). From this result, it is expected that the CS mutant lacks protease activity but retains ubiquitin-binding ability. Therefore, the observed difference in co-immunoprecipitated ubiquitinated proteins implies that CAPN15 recognizes ubiquitinated proteins as substrates, that is, it functions as a ubiquitin-directed protease.

### CAPN15 is involved in the regulation of cell morphology

**Figure 2:**
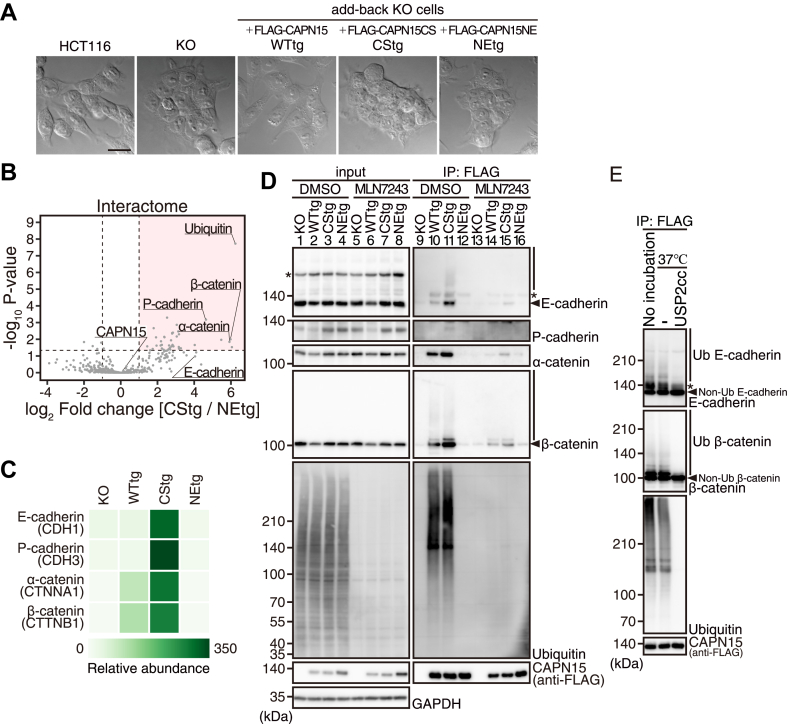
**CAPN15 recognizes the ubiquitinated cadherin-catenin complex *via* the NZF domains.***A*, differential interference contrast images of HCT116, KO, WTtg, CStg, and NEtg cells. Scale bar, 20 μm. *B*, the mean log_2_ fold change of the protein abundance (CStg *versus* NEtg immunoprecipitates) with -log_10_*p* value of the interactome was shown on the x- and y-axes, respectively. Significantly increased proteins were defined as those with the value of the log_2_ fold change >1 with *p* value < 0.05 (plots in a colored area). It should be noted that there was no change in abundance of CAPN15 in the CStg and NEtg immunoprecipitates. *C*, the heatmap shows the relative abundance of cadherins and catenins in the KO, WTtg, CStg, and NEtg immunoprecipitates. *D*, KO, WTtg, CStg, and NEtg cells were treated with dimethyl sulfoxide (DMSO; vehicle) or 1 μM MLN7243. Cell lysates were subjected to immunoprecipitation using an anti-FLAG antibody, followed by western blotting. *Asterisks* indicate nonspecific bands. *Vertical lines* indicate the ubiquitinated forms. *E*, immunoprecipitated fraction from DMSO (vehicle)-treated CStg cells was incubated with or without ubiquitin-specific protease 2 (USP2cc), followed by western blotting. The *asterisk* indicates the non-specific band. *Vertical lines* indicate the ubiquitinated forms.

To investigate the function of CAPN15, we generated HCT116 cells in which the *CAPN15* gene was knocked out (KO cells; Fig. 2*A*). To test whether CAPN15 function overlaps with that of the proteasome, we investigated the effect of *CAPN15* KO on the processing of ubiquitinated proteins (Fig. S2*A*). The results showed that, when compared to HCT116 cells, the levels of ubiquitinated proteins did not change in KO cells treated with or without bortezomib, a proteasome inhibitor, indicating that CAPN15 does not overlap with the proteasome in the bulk degradation of ubiquitinated proteins. Intriguingly, we found that KO cells congregated more tightly than HCT116 cells (Fig. 2*A*; HCT116 *versus* KO). This phenotype was rescued by the stable addition of FLAG-WT, but not FLAG-CS or FLAG-NE (WTtg, CStg, and NEtg cells, respectively; Fig. 2*A*; see Table S1 for abbreviations). WTtg cells tended to be more scattered than HCT116 cells (Fig. 2*A*; WTtg *versus* HCT116). Western blot analysis showed that the expression levels of FLAG-WT, FLAG-CS, and FLAG-NE in WTtg, CStg, and NEtg cells, respectively, were much higher than that of endogenous CAPN15 (Fig. S2*B*). Therefore, we reasoned that the morphology of WTtg cells was caused by excessive CAPN15 protease activity. These observations suggested that both the protease activity and the ubiquitin-binding ability of CAPN15 contribute to cell morphology.

### CAPN15 recognizes the ubiquitinated cadherin-catenin complex *via* the NZF domain

Next, we investigated the molecular mechanism underlying KO cell morphology through interactome analysis of CAPN15. Immunoprecipitated fractions prepared using anti-FLAG antibodies from KO, WTtg, CStg, and NEtg cells were processed by liquid chromatography-mass spectrometry and intensity-based label-free quantification with the biological duplicates (Fig. S2*C*). A total of 400 proteins were identified (Table S2).

As shown in Figure 1*C*, ubiquitinated proteins bound *via* the ZF domains were more abundant in FLAG-CS than in FLAG-WT. Therefore, to identify the proteins that interact with CAPN15 *via* the ZF domains, we compared the immunoprecipitates from CStg and NEtg cells. This analysis identified 42 proteins whose relative abundances in the CStg immunoprecipitate were increased by more than two-fold (log_2_ fold change >1) (*p*-value <0.05; Figure 2*B*, plots in a colored area). Most of these proteins were components of the ubiquitin–proteasome system. These interactions are likely to reflect non-specific binding under the experimental conditions and therefore were not further pursued. Among the remaining proteins, P-cadherin/CDH3, α-catenin/CTNNA1, and β-catenin/CTNNB1, components of the cadherin-catenin complex that play a crucial role in cell adhesion (21, 22, 23), were increased significantly (more than 16-fold) in the CStg immunoprecipitate. E-cadherin/CDH1 was also increased to a similar extent, although the *p*-value did not reach significance (*p* > 0.05; Fig. 2, *B* and *C*). Another component of the cadherin-catenin complex, p120-catenin, which is responsible for stabilizing the complex at the cell surface (24, 25, 26), was not identified in any of the immunoprecipitates (Table S2).

Western blot analysis was performed to confirm the results of the interactome analysis. Except for P-cadherin, the expression levels of E-cadherin and catenins were higher in KO, CStg, and NEtg cells compared to HCT116 and WTtg cells (Figs. 2*D*, lanes 1–4, 4*A*, short-exposure panels, and S3). Consistent with the interactome analysis, these cadherins and catenins were more abundantly immunoprecipitated using anti-FLAG antibodies from CStg cells than from WTtg cells, but not from NEtg cells (Fig. 2*D*, lanes 9–12). For E-cadherin and β-catenin, ubiquitinated forms were also detected as high molecular weight smears (Fig. 2*D*, lanes 10 and 11), since they were degraded by ubiquitin-specific protease 2 which cleaves all conventional ubiquitin-conjugations (Fig. 2*E*). In addition, inhibition of ubiquitination by the treatment of these cells with MLN7243, a ubiquitin-activating enzyme (E1) inhibitor, reduced the anti-FLAG immunoprecipitation of cadherins and catenins from WTtg and CStg cells without affecting their original expression levels (Fig. 2*D*, lanes 5–8 and 13–16). These results identified E- and P-cadherins and α- and β-catenins as ubiquitination-dependent CAPN15-interacting proteins.

**Figure 3:**
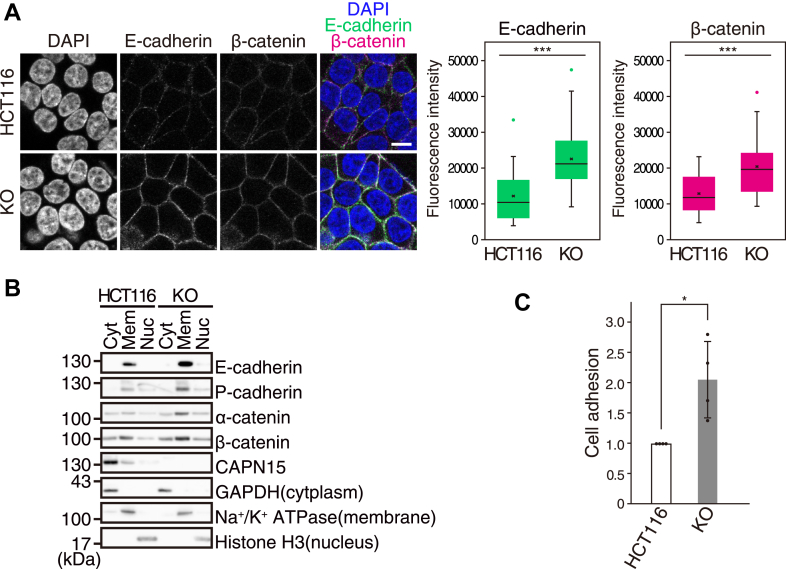
**CAPN15 deficiency enhances E-cadherin-mediated cell adhesion.***A*, immunofluorescence of E-cadherin and β-catenin in HCT116 and KO cells. This scale bar represents, 10 μm. Fluorescence intensities of E-cadherin and β-catenin were quantified and are presented as box plots showing the median (*line*), mean (×), interquartile range (*box*), and minimum/maximum values (*whisker*). Outliers are indicated as individual dots. ∗∗∗*p* < 0.001 by Welch’s *t* test; n = 40 for HCT116 cells and n = 47 for KO cells. *B*, HCT116 and KO cells were fractionated into cytoplasm (Cyt), membrane (Mem), and nucleus (Nuc), and the fractions were analyzed by western blotting. Na^+^/K^+^-ATPase, GAPDH, and Histone H3 are markers of membrane, cytoplasmic, and nuclear fractions, respectively. *C*, Cell adhesion assay. HCT116 and KO cells were seeded onto E-cadherin-Fc-coated plates and incubated for 15 min. After washing away nonadherent cells, the remaining adherent cells were fixed and stained with crystal violet. Absorbance was measured at 595 nm as an index of cell adhesion, and the values were normalized to that of HCT116 cells, which was defined as 1. Data are presented as the mean ± SD with individual data points (n = 4). ∗*p* < 0.05 by Wilcoxon rank-sum test.

E- and P-cadherins belong to the classical cadherin family of transmembrane glycoproteins that have been shown to play crucial roles in cell adhesion. The extracellular region of the cadherins mediates Ca^2+^-dependent homophilic interactions, and the cytoplasmic region form a stable complex with p120-, α-, and β-catenins. p120-catenin binds to the juxtamembrane domain (JMD; see Fig. S5*A*) to inhibit cadherin endocytosis, and β-catenin binds to the cadherin binding domain to anchor the complex to the actin cytoskeleton through α-catenin (25, 26, 27, 28). We compared the expression of cadherins and catenins in HCT116 and KO cells. As expected, increased expression of E-cadherin and β-catenin at the cell surface of KO cells was observed by immunofluorescence (Fig. 3*A*). Cell fractionation analysis showed increased expression of E- and P-cadherins and α- and β-catenins in membrane fraction of KO cells (Fig. 3*B*). In addition, we quantitatively measured cell adhesion using E-cadherin-Fc-coated plates. KO cells exhibited significantly increased adhesion to E-cadherin compared with HCT116 cells (Fig. 3*C*), consistent with the increased expression of E-cadherin at the cell surface. Together, these results prompted us to speculate that CAPN15 recognizes ubiquitinated cadherin-catenin complexes to cleave one or more of their components, thereby preventing their accumulation at the cell surface.

### CAPN15 cleaves E-cadherin in a Ca^2+^- and ubiquitination-dependent manner

**Figure 4:**
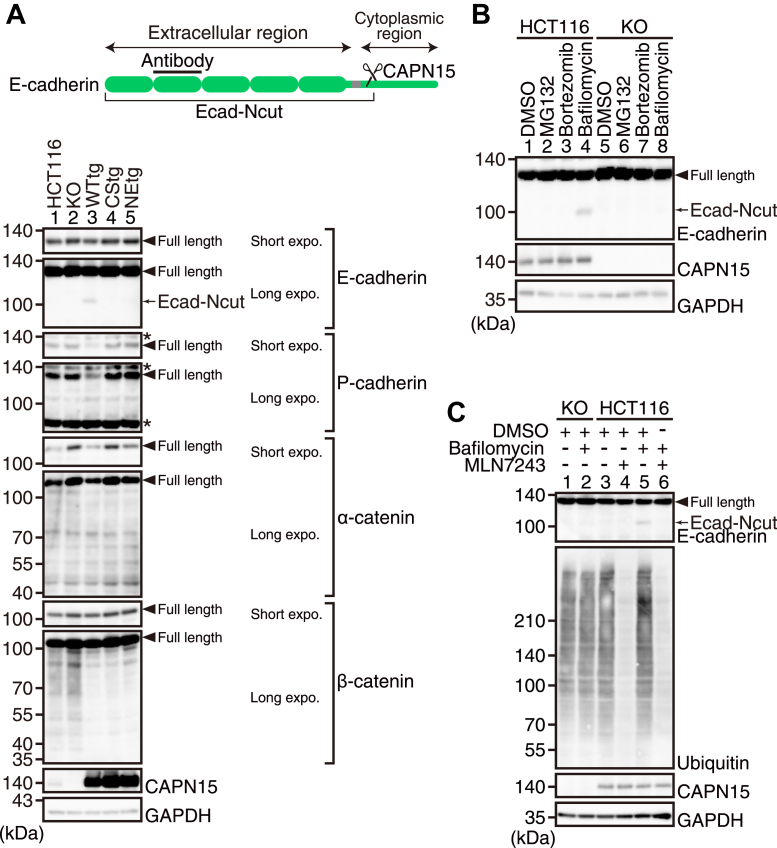
**CAPN15 mediates E-cadherin cleavage, leading to lysosomal degradation of the resultant fragment.***A*, whole cell lysates from HCT116, KO, WTtg, CStg, and NEtg cells were analyzed by western blotting. For E- and P-cadherins and α- and β-catenins, short and long exposed blots are shown. *Asterisks* indicate non-specific bands. Schematic of the E-cadherin domain structure with anti-E-cadherin antibody recognition site and a predicted cleavage site is also shown. *B*, After HCT116 and KO cells were treated with DMSO (vehicle), 10 μM MG132, 1 μΜ bortezomib, or 100 nM bafilomycin A1, the cell lysates were analyzed by western blotting. *C*, HCT116 and KO cells were treated with DMSO (vehicle), 1 μΜ MLN7243, and 100 nΜ bafilomycin A1 as indicated. After the treatment, the cell lysates were analyzed by western blotting.

To determine the substrate for CAPN15, Western blot analysis was performed on cell lysates from HCT116, KO, WTtg, CStg, and NEtg cells cultured under normal conditions. The results showed that a 100-kDa N-terminal fragment of E-cadherin (Ecad-Ncut) was faintly detected in WTtg cells using an anti-E-cadherin antibody against the extracellular domain (Fig. 4*A*, lane 3). No cleavage products of β-catenin were detected under these conditions, suggesting that β-catenin is not a substrate for CAPN15. Because Ecad-Ncut was barely detectable in HCT116 cells, we hypothesized that Ecad-Ncut was rapidly degraded under these conditions. To identify the proteolytic pathway responsible for Ecad-Ncut degradation, HCT116 cells were treated with proteasome and lysosome inhibitors. As shown in Figure 4*B*, Ecad-Ncut was detected in HCT116 cells treated with bafilomycin A1, a lysosome inhibitor (lane 4). Furthermore, Ecad-Ncut detected in bafilomycin A1-treated HCT116 cells was abrogated in the presence of MLN7243 (Fig. 4*C*, lanes 5 and 6). These results suggested that E-cadherin is a substrate for CAPN15, and that Ecad-Ncut rapidly undergoes lysosomal degradation.

**Figure 5:**
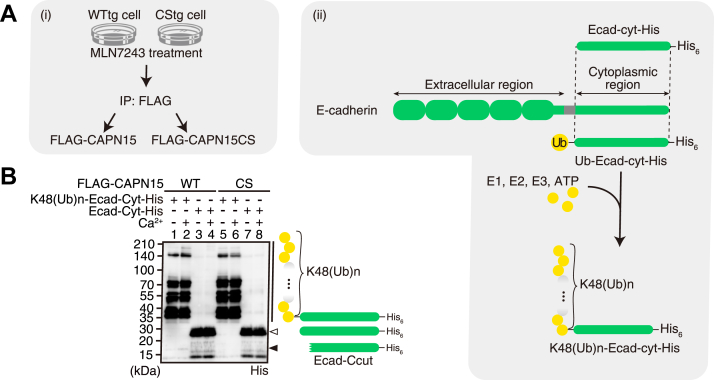
**CAPN15 cleaves E-cadherin in a Ca^2+^- and ubiquitin-dependent manner *in vitro*.***A*, preparation of recombinant CAPN15 and E-cadherin substrates. (i) FLAG-CAPN15 and FLAG-CAPN15CS were immunopurified with anti-FLAG antibody from WTtg and CStg cells, respectively. To prevent co-purification of CAPN15-bound ubiquitinated proteins, these cells were treated with MLN7243 before purification. (ii) Ecad-cyt-His, and N-terminally monoubiquitin-tagged Ecad-cyt-His_6_ (Ub-Ecad-cyt-His) were purified through a Ni^2+^-affinity column. Ub-Ecad-cyt-His was then incubated with E1, gp78-UBE2G2 (E2-E3 chimera), ubiquitin, and ATP to elongate K48-linked ubiquitin chains. The resultant substrate, K48(Ub)n-Ecad-cyt-His, was purified through a Ni^2+^-affinity column to remove free ubiquitin. For details, see also Experimental Procedures section. *B*, K48(Ub)_n_-Ecad-cyt-His or Ecad-cyt-His was incubated with FLAG-CAPN15 (WT) or FLAG-CAPN15CS (CS) in the absence (−) or presence (+) of Ca^2+^ as indicated. The reactions were analyzed by western blotting using anti-His antibodies. The closed *arrowhead* indicates a cleaved fragment (Ecad-Ccut), which was increased in the presence of Ca^2+^ (lane 2) compared with the absence of Ca^2+^ (lane 1).

Next, we reconstituted E-cadherin cleavage by CAPN15 *in vitro* using recombinant CAPN15 and E-cadherin. To investigate the effect of the ubiquitin-binding ability of CAPN15, we prepared C-terminally His_6_-tagged E-cadherin cytoplasmic regions with or without an N-terminal K48-linked polyubiquitin tag, K48(Ub)_n_-Ecad-cyt-His and Ecad-cyt-His, respectively, as substrates (Figs. 5*A* and S4; see Table S1 for abbreviations). When the substrates were incubated with FLAG-CAPN15, K48(Ub)_n_-Ecad-cyt-His, but not Ecad-cyt-His, was cleaved to produce a C-terminal fragment of approximately 17 kDa (Ecad-Ccut), and this cleavage was enhanced in the presence of Ca^2+^ (Fig. 5*B*, lanes 2 *versus* lane 1, closed arrowhead). In contrast, FLAG-CAPN15CS failed to generate the Ecad-Ccut fragment (lanes five and 6). The faint Ecad-Ccut band detected in the absence of Ca^2+^ (lane 1) was probably due to the activity of the Ca^2+^-bound form of FLAG-CAPN15 which was co-purified with the Ca^2+^-free form, although the possibility cannot be excluded that the CAPN15 activity is Ca^2+^-independent. Thus, we concluded that CAPN15 cleaves E-cadherin in a Ca^2+^ and ubiquitination-dependent manner.

### CAPN15 cleaves E-cadherin within the juxtamembrane domain in the absence of p120-catenin

The apparent size of Ecad-Ccut suggested that the E-cadherin cleavage occurred within the JMD (aa. 734–779) (Fig. S5*A*). To determine the cleavage site, E-cadherin deletion mutants shown in Fig. S5*A* were co-expressed with FLAG-CAPN15 in KO cells and analyzed by western blotting (Fig. S5*B*). Deletion of the central region of the JMD abolished CAPN15-mediated cleavage; mutants 3, 4, and 7 were not cleaved, whereas mutants 8 and 9 were barely cleaved. These results indicated that CAPN15 cleaves E-cadherin within residues 754 to 759 (Fig. S5*B*, mutant 7).

**Figure 6:**
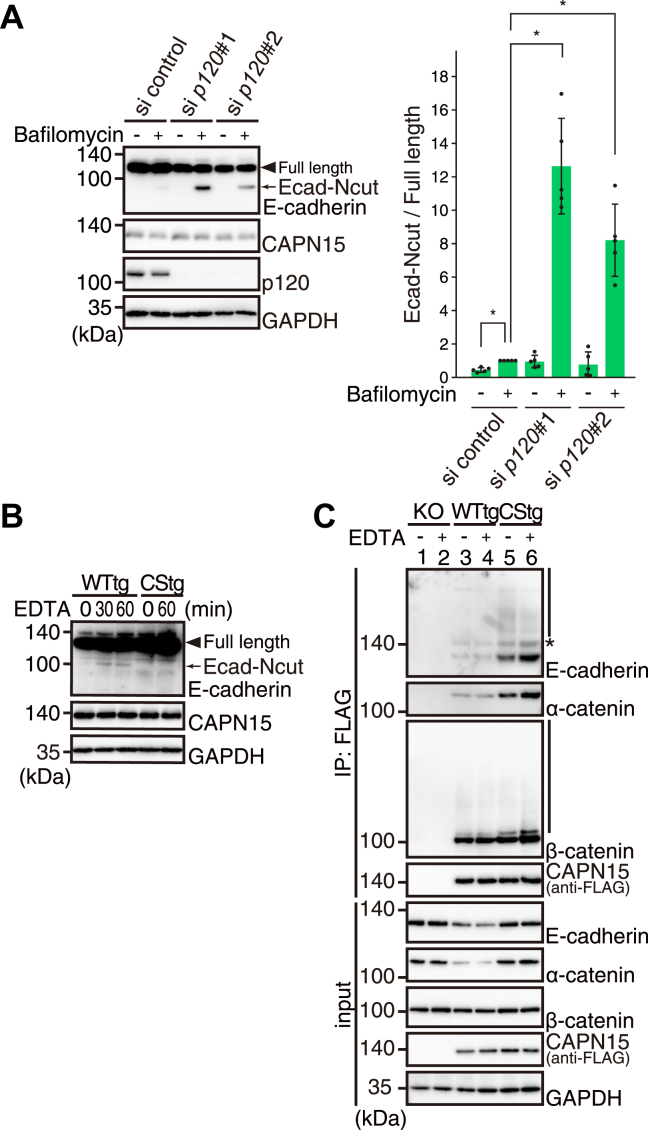
**CAPN15 cleaves E-cadherin in the absence of p120-catenin and upon loss of cell adhesion.***A*, (*left*) HCT116 cells were transfected with control or p120-catenin siRNAs. Forty-eight hours after the transfection, cells were treated with DMSO (−) or bafilomycin A1 (+) for 4h. The cell lysates were subjected to western blotting. (*right*) Quantification of Ecad-Ncut relative to the full-length E-cadherin. The values were normalized to the relative intensity in bafilomycin A1-treated HCT116 cells transfected with control siRNA, which was defined as 1. The graph represents the mean ± SD with individual data points (n = 5). ∗*p* < 0.05 by Steel-Dwass test. *B*, after WTtg and CStg cells were treated with 2 mM EDTA for the indicated periods, the cell lysates were analyzed by western blotting. *C*, KO, WTtg, and CStg cells were treated with (+) or without (−) 2 mM EDTA for 30 min. The cell lysates were subjected to immunoprecipitation using anti-FLAG antibodies, followed by western blotting. The *asterisk* indicates a nonspecific band. *Vertical lines* indicate the ubiquitinated forms.

Since this region overlaps with the p120-catenin binding domain, we examined whether the absence of p120 enhances CAPN15-mediated E-cadherin cleavage. Upon p120-catenin knockdown, the level of Ecad-Ncut increased significantly, despite the unchanged expression levels of CAPN15 (Fig. 6*A*). This suggests that p120-catenin binding protects E-cadherin from cleavage by CAPN15, likely by sterically masking the cleavage site. Previous studies have shown that dissociation of p120-catenin from the cadherin-catenin complex at the cell surface triggers rapid internalization of cadherin (26). Therefore, it is likely that CAPN15 cleaves E-cadherin at or near the plasma membrane, specifically during the brief period between p120-catenin dissociation and the onset of endocytosis.

### CAPN15 cleaves E-cadherin upon loss of cell adhesion

E-cadherin is dynamically regulated through endocytic, recycling, or proteolytic pathways depending on cellular context (29). To investigate the cellular conditions under which CAPN15-mediated cleavage of E-cadherin is triggered, we treated cells with EDTA to chelate extracellular Ca^2+^ and disrupt Ca^2+^-dependent cadherin–cadherin interactions, thereby weakening epithelial cell–cell adhesion (30, 31). Notably, EDTA treatment increased Ecad-Ncut levels in WTtg cells, but not in CStg cells (Fig. 6*B*). Concomitantly, EDTA treatment enhanced the anti-FLAG immunoprecipitation of E-cadherin and catenins in CStg cells (Fig. 6*C*, lanes 5 and 6). These results suggested that CAPN15 targets E-cadherin molecules that must be cleared from the plasma membrane once their adhesive function is abrogated.

### Loss of CAPN15 results in E-cadherin accumulation in mouse epithelial tissues

**Figure 7:**
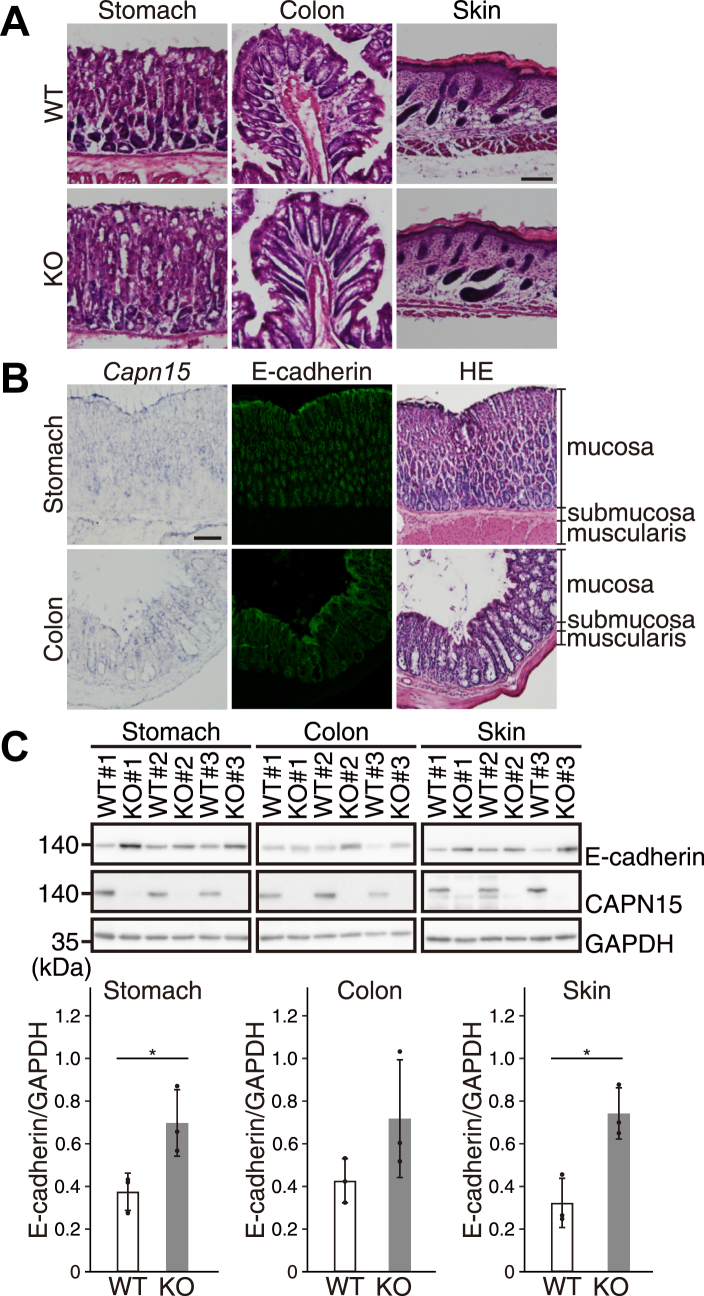
**E-cadherin is accumulated in epithelial tissues of *Capn15* KO mice.***A*, hematoxylin-eosin images of the stomach (*left*), colon (*middle*), and skin (*right*) from WT and *Capn15* KO (KO) mice. This scale bar represents, 100 μm. *B*, serial sections of mouse stomach (*upper panels*) and colon (*lower panels*) were subjected to *in situ* hybridization for *Capn15* (*left*), immunofluorescence for E-cadherin (*middle*), and hematoxylin-eosin staining (*right*). This scale bar represents, 100 μm. *C*, protein extracts from the stomach, colon, and skin of three pair (#1–3) of WT and *Capn15* KO (KO) littermates were analyzed by western blotting. The E-cadherin levels were quantified and normalized by GAPDH. The graph represents the mean ± SD with individual data points (n = 3). ∗*p* < 0.05 by Welch’s *t* test.

The *in vivo* relevance of E-cadherin cleavage by CAPN15 was investigated using *Capn15* KO mice generated by the CRISPR-Cas9 system (Fig. S6*A*). Consistent with previous reports (10, 14, 15), our KO mice exhibited ocular and brain abnormalities as well as growth retardation. We next analyzed representative epithelial tissues such as the stomach, colon and skin, since these tissues express high levels of E-cadherin. No overt abnormalities were observed in these tissues from KO mice, suggesting that the lack of CAPN15 is tolerated in terms of epithelial integrity *in vivo* (Fig. 7*A*). However, to assess potential molecular changes that might not be reflected morphologically, we further examined whether CAPN15 activity and the expression of E-cadherin are inversely related in these tissues, as observed in HCT116 cells. In WT mice, overlapping expression of *Capn15* and E-cadherin was confirmed in the mucosal epithelium of the stomach and colon by *in situ* hybridization and immunofluorescence, respectively (Fig. 7*B*). Western blot analysis of the stomach, colon, and skin homogenates from WT and KO littermates revealed increased E-cadherin protein levels in KO mice, with a similar but not statistically significant trend observed in the colon (Fig. 7*C*). Notably, no significant changes were detected at the mRNA level (Fig. S6*B*), indicating that the observed change occurs at the protein level. These results demonstrated that CAPN15 is involved in the regulation of E-cadherin *in vivo*.

## Discussion

Although most NZF-containing proteins are involved in ubiquitin-related pathways, the function of CAPN15, which contains two tandem NZF domains, has remained unexplored. In this study, we demonstrated that CAPN15 is a non-proteasomal, ubiquitin-directed protease that proteolytically regulates cell surface E-cadherin levels by recognizing the ubiquitinated E-cadherin-catenin complex. Previous studies have reported that DDI2 (Ddi1 in yeast), a ubiquitin shuttling factor that brings ubiquitinated proteins to the proteasome, also exhibits a ubiquitin-directed protease activity that complements the proteasome activity (32, 33). In contrast, CAPN15 does not contribute to proteasome-like bulk degradation of ubiquitinated proteins, as the loss of CAPN15 did not result in the accumulation of ubiquitinated proteins, which is typically observed upon proteasome inhibition (Fig. S2*A*). Consequently, this study unveiled a ubiquitin-related proteolytic system distinct from the ubiquitin-proteasome system.

According to the commonly accepted notion, calpains recognize the three-dimensional or higher-order structures rather than specific amino acid sequences or post-translational modifications of their substrates (34, 35). However, our findings reveal that ubiquitination serves as an indispensable post-translational modification for substrate recognition by CAPN15, highlighting an unexpected layer of regulation that contributes to substrate specificity among the calpain family members.

A key question arose regarding whether the substrate specificity of CAPN15 depends on the length or types of the attached ubiquitin chains. Our pull-down assay showed that the NZF domains of CAPN15 preferred longer ubiquitin chains to mono- and di-ubiquitins (Fig. S1). Tandemly arranged NZF domains contribute to the binding of longer ubiquitin chains by providing additional binding sites, as demonstrated for TRABID, a deubiquitinating enzyme containing three NZF domains (36). A similar mechanism could apply to CAPN15; however, it appears that CAPN15 shows no strict preference for the length of polyubiquitin chains. In addition, the architecture of polyubiquitin chains is diverse, formed through one or more of the eight distinct linkage types, each of which provides a specific signal that regulates various cellular processes (37). Michel *et al.* reported that most NZF domains, including the ZF1 and ZF2 domains of CAPN15, do not exhibit apparent linkage specificity. However, some of these, including the CAPN15 ZF1 domain, contain secondary patches conserved across species that may recognize substrate interfaces near their ubiquitination site (18). It is proposed that a dual binding mechanism between ubiquitinated substrate and NZF domains may explain why ubiquitin-dependent protein-protein interactions could be specific. Since CAPN15 recognizes the E-cadherin-catenin complex in which E-cadherin and β-catenin are ubiquitinated (Fig. 2), it is plausible that this recognition could be explained by this dual binding mechanism between ubiquitinated E-cadherin and the ZF1 domain: ubiquitin chain binds to the TF motif and E-cadherin binds to the secondary patch near the ubiquitination site. In addition, ubiquitin binding to the ZF1 and ZF2 domains may induce structural modifications that enable E-cadherin cleavage, as CAPN15 does not cleave the E-cadherin fragment lacking the ubiquitin-tag even in the presence of Ca^2+^ (Fig. 5*B*). We also found that the ubiquitinated proteins immunoprecipitated with the full-length CAPN15 constructs (WT and CS) were less abundant than those immunoprecipitated with the ZF construct (Fig. 1*C*), thus suggesting that non-ZF domains, such as the SOL-homologous domain, may also contribute to the selective recognition of substrates.

**Figure 8:**
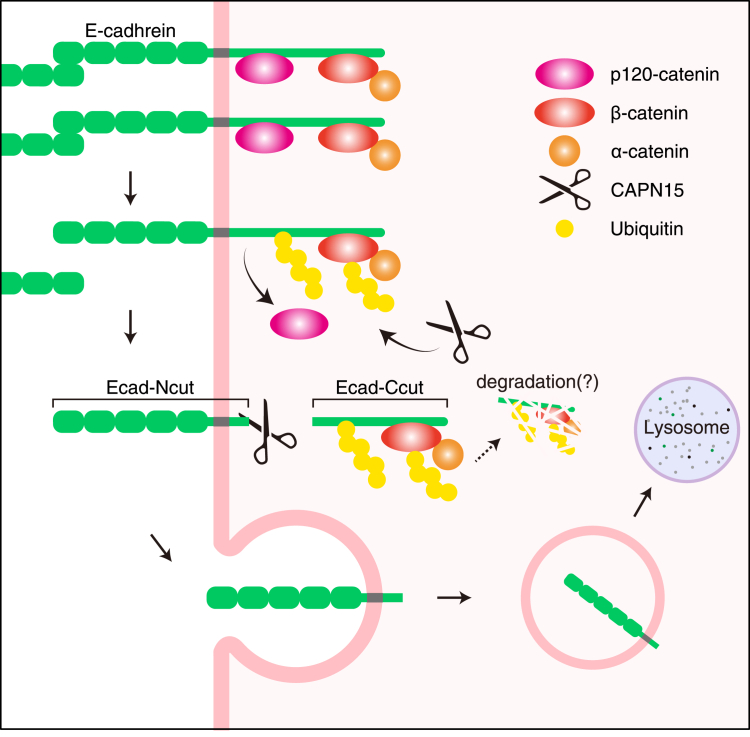
**Proposed model. Under certain physiological conditions, such as a localized decline in extracellular Ca^2+^, homophilic interactions of E-cadherin are disrupted.** The affected E-cadherin-catenin complexes then undergo specific ubiquitination, accompanied by the dissociation of p120-catenin, and are subsequently cleaved by CAPN15. The resultant Ecad-Ncut fragment is rapidly internalized and degraded in the lysosome. Other cleavage products, including the Ecad-Ccut fragment and catenins are also presumed to be degraded, although the responsible proteolytic systems remain unidentified.

There are approximately 20 structurally conserved classical cadherins, including E- and P-cadherins (38). These cadherins form complexes with catenins through their cytoplasmic domains to anchor the actin cytoskeleton, and organize adherens junctions in various tissues. Adherens junctions undergo continuous formation, dissolution, and reorganization, depending on changes in cell surface cadherin levels. In various cell lines, E-cadherin is downregulated by intracellular and extracellular proteases, leading to reduced cell adhesion (39, 40, 41, 42, 43, 44, 45). In this study, we found that CAPN15 modulates the dynamics of E-cadherin by recognizing ubiquitinated E-cadherin-catenin complex, thereby acting as a regulator of cell adhesion. As illustrated in Figure 8, cells occasionally encounter specific conditions that disrupt E-cadherin homophilic interactions. Under such conditions, the affected E-cadherin-catenin complexes undergo ubiquitination following the dissociation of p120-catenin (24, 46). Concomitantly, intracellular Ca^2+^ levels increase and CAPN15 is recruited to the ubiquitinated complexes to cleave E-cadherin. The resulting Ecad-Ncut is internalized *via* endocytosis and targeted for lysosomal degradation, likely due to its C-terminal di-leucine motif, which is known to mediate E-cadherin internalization (Fig. S5*A*) (26).

In this model, the following points remain unclear. One is the E3 ubiquitin ligase of the E-cadherin-catenin complex, specifically that of E-cadherin. In the ubiquitination process, E3 ubiquitin ligases play a crucial role in transferring ubiquitin to specific target proteins. However, neither individual nor combined knockdown of four E3 ubiquitin ligases that have been reported to act on E-cadherin or β-catenin suppressed E-cadherin cleavage by CAPN15 (Fig. S7) (47, 48, 49, 50, 51). These results suggest that functional redundancy among the tested E3 ligases is unlikely, and point to the involvement of other, yet unidentified, E3 ligases responsible for this process. Another point is the fate of the C-terminal cleavage fragment of E-cadherin (Ecad-Ccut) and the bound α- and β-catenins. So far, no apparent function has been described for the C-terminal E-cadherin fragments generated by other proteases. Regarding catenins, cytosolic β-catenin is known to translocate into the nucleus where it functions as a transcriptional regulator in the Wnt signaling pathway (52). In our analysis, however, the levels of α- and β-catenins in the cytosol and nucleus fractions of HCT116 cells were not accumulated compared to KO cells (Fig. 3*B*), suggesting further degradation. Further studies are required to fully elucidate these downstream signaling pathways by which CAPN15 regulates E-cadherin expression.

In our *in vitro* reconstitution system, CAPN15 cleaved ubiquitinated E-cadherin with relatively low stoichiometry. This likely reflects the absence of cellular cofactors that facilitate the reaction, such as catenins that form a complex with E-cadherin, and/or partial loss of CAPN15 activity during purification. Indeed, knockdown of p120 catenin in cells was associated with a substantial increase in Ecad-Ncut, suggesting that the cellular context, including complex formation and other post-translational modifications, may enhance CAPN15-mediated cleavage. These findings indicate that CAPN15 activity depends on the structural and biochemical environment of its substrates within the cell.

Our *in vivo* analysis showed that *Capn15* KO mice exhibited developmental abnormalities such as ocular defects and growth retardation, but no overt phenotypes in the stomach, colon, and skin (Fig. 7*A*). However, E-cadherin levels were moderately increased in KO mice, although the increase in the colon did not reach statistical significance. We attribute these modest changes to functional redundancy among calpains, particularly, compensatory action of calpain-1 and calpain-2, which are expressed in epithelial tissues and have been reported to proteolytically regulate E-cadherin (42). Thus, while CAPN15 dominantly contributes to the regulation of cadherin stability in cultured cell lines, the effect of its absence can be masked *in vivo* by compensatory proteolytic pathways.

These mechanistic insights raise important questions about the physiological roles of CAPN15 in tissue development and disease. Recent reports have indicated that the loss of CAPN15 function leads to congenital anomalies in both humans and mice, highlighting its prenatal role (10, 11, 12, 14, 15). Our findings have shown that the absence of CAPN15 resulted in the accumulation of E-cadherin, both in cultured cells and *in vivo*. E-cadherin, along with other classical cadherins, plays a crucial morphogenetic role by mediating cell-cell interactions necessary for cell sorting, cell movements, cell polarity, and boundary formation. However, high levels of E-cadherin disrupt these biological processes (53, 54, 55, 56). Therefore, it is plausible that CAPN15 regulates E-cadherin expression to maintain the balance required for proper developmental morphogenesis and epithelial homeostasis.

Furthermore, our findings also suggest a broader role for CAPN15 in regulating other classical cadherins beyond E-cadherin. We identified P-cadherin, another classical cadherin, as a binding partner and potential substrate of CAPN15, suggesting that the mechanism by which CAPN15 and ubiquitin regulate E-cadherin expression may extend to other classical cadherins as well. In the developing brain, where classical cadherins play essential roles in processes such as neural compartmentalization, neuroblast migration, axon pathfinding, and synapse formation, the loss of CAPN15 function causes significant phenotypes in mice (57, 58), raising the possibility that the neurological symptoms observed in OGIN patients may result from the dysregulation of multiple classical cadherins caused by CAPN15 deficiency.

It is also possible that CAPN15-mediated cleavage of cadherins induces only modest changes in the cellular proteome, which may explain why cadherins or related molecules were not identified in the proteomic screening of the developing mouse brain (59). Notably, this study did not take into account the role of ubiquitination in substrate identification. Therefore, future investigations into the CAPN15–classical cadherin axis should consider the ubiquitin system as an additional layer of regulation, which may provide deeper insights into CAPN15’s functions and its involvement in OGIN pathogenesis.

In summary, we identified CAPN15 as a non-proteasomal, ubiquitin-directed protease that selectively regulates E-cadherin and may also act on other classical cadherins. Our findings uncover a previously unappreciated proteolytic mechanism and establish CAPN15 as a critical regulator of epithelial homeostasis and developmental morphogenesis.

## Experimental procedures

### Plasmid construction

All of the plasmid constructs were verified by DNA sequencing. Amplification of cDNA fragments was performed using KOD-Plus-Neo DNA polymerase (Toyobo) unless otherwise specified. For protein expression in cultured cells, cDNAs were cloned into modified pcDNA3.1 (Invitrogen) to produce proteins with an N-terminal FLAG tag or a C-terminal myc tag. The CAPN15 point mutants, and E-cadherin in-frame deletion mutants were generated by PCR-mediated site-directed mutagenesis using the Pfu Turbo DNA polymerase (Agilent). For *in vitro* ubiquitin-binding assay, cDNAs encoding budding yeast Rpn10 and full-length and truncated human CAPN15 ZF region were amplified by PCR and cloned into pGEX-6P-1 (Cytiva). For *in vitro* cleavage assay, cDNAs encoding human ubiquitin G76V-fused human E-cadherin cytoplasmic region, and gp78RING-Ube2g2 (a chimera of the RING domain from E3 ubiquitin ligase gp78 and ubiquitin-conjugating enzyme Ube2g2) (60) were amplified by PCR and cloned into pET26b (Merck Millipore) and pGEX-6P-1, respectively.

Plasmids were constructed for gene editing using the transcription activator-like effector nuclease (TALEN) method. Site-specific TALEN plasmids for *CAPN15* and adeno-associated virus integration site 1 (AAVS1) loci were constructed as described previously (61). The set of the plasmids used in this study, TALE monomer template plasmids (Addgene #32180-32183) and TALEN backbone plasmids (Addgene #32189-32192), were gifted by Feng Zhang (61). A donor plasmid for generating KO cells, pBluescript-CAPN15KO, was composed of an approximately 1000 bp of upstream and downstream sequences flanking the targeted site within the first coding exon (exon 4) of *CAPN15* (Ensembl gene ID: ENSG00000103326), with the puromycin resistance gene inserted into pBluescript (Agilent). Donor plasmids pZDonor-AAVS1-FLAG-CAPN15, pZDonor-AAVS1-FLAG-CAPN15CS, and pZDonor-AAVS1-FLAG-CAPN15NE were constructed for generating WTtg, CStg, and NEtg cells, respectively. Briefly, the EF1α promoter, PCR-amplified FLAG-CAPN15, FLAG-CAPN15CS, and FLAG-CAPN15NE cDNAs, and SV40-polyA were tandemly inserted into pZDonor-AAVS1-Neo, in which the puromycin resistance gene of pZDonor-AAVS1-puromycin (Sigma-Aldrich) was replaced with the neomycin resistance gene.

### Generation of *CAPN15* KO cells

*CAPN15* KO cells were generated using TALEN-mediated gene editing. pBluescript-CAPN15KO was transfected with *CAPN15* site-specific TALEN plasmids into HCT116 cells (ATCC) using a NEPA21 electroporator (NEPAGENE). Puromycin-resistant clones were isolated, and *CAPN15* gene disruption was validated by DNA sequencing and western blotting.

### Generation of WTtg, CStg, and NEtg cells

WTtg, CStg, and NEtg cells were generated using TALEN-mediated gene integration into the AAVS1 locus. pZDonor-AAVS1-FLAG-CAPN15, pZDonor-AAVS1-FLAG-CAPN15CS, and pZDonor-AAVS1-FLAG-CAPN15NE were transfected with AAVS1 site-specific TALEN plasmids using the NEPA21 electroporator. Neomycin-resistant clones were isolated and validated by PCR and western blotting.

### Generation of *Capn15* KO mice

All of the experimental animal procedures were approved by the Animal Use and Care Committee of the Tokyo Metropolitan Institute of Medical Science (approval number: 24–034), and the animals were treated according to the committee’s guidelines. All the mice were housed in specific pathogen-free facilities at our institute in individually ventilated cages under controlled conditions (23 ± 1°C, 50 ± 10% humidity, 12 h light/dark cycle), with *ad libitum* access to water and standard chow (CE-2, CLEA Japan Inc.). C57BL/6N mice were purchased from CLEA Japan Inc. *Capn15* KO mice were generated using the CRISPR-Cas9 system. A 19-nt target sequence in exon 2 with a protospacer-adjacent motif, 5′-agatctggcttatgtcggg(ggg)-3′, was selected using CRISPRdirect (http://crispr.dbcls.jp/) to introduce a frameshift mutation early in the coding sequence. The *Capn15* guide RNA was generated using MEGAshortscript T7 kit (Thermo Fisher Scientific) and a DNA template containing the T7 promoter and the target sequence, and purified using MEGAclear kit (Thermo Fisher Scientific). *Cas9* mRNA (10 ng/μl) and the *Capn15* guide RNA (5 ng/μl) were microinjected into the cytoplasm or prenucleus of fertilized eggs collected from frozen pronuclear-stage C57BL/6N mouse embryos (ARK Resource) using a micromanipulator system (Leica Microsystems). Injected embryos were transferred into pseudopregnant Crlj:ICR-nu (nu/+) females (Jackson Laboratory Japan Inc.) to obtain heterozygous or homozygous offspring. *Capn15* KO mice used in this study had a 23-bp deletion, resulting in a premature stop codon at the 94th codon. Genotyping was performed by PCR with primers flanking the deletion site, 5′-gccacagttggggagtggt-3′ and 5′-cacacttcgcaagcctc-3′, under the following conditions: 94 °C for 3 min; 30 cycles of 94 °C for 30 s, 60 °C for 30 s, and 72 °C for 3 min; followed by a hold at 4 °C. Loss of CAPN15 protein expression in KO mice was confirmed by western blotting.

### SDS-PAGE and western blotting

Protein samples were mixed with SDS-PAGE sample buffer (50 mM Tris-HCl, pH 6.8, 1.6% SDS, 0.02% bromophenol blue, 8% glycerol, 20 mM DTT) and heated for 5 min at 95 °C, or NuPAGE LDS sample buffer (Thermo Fisher Scientific) and heated for 10 min at 70 °C. Proteins were separated by SDS-PAGE on 5 to 10% or 7.5 to 15% gradient precast gels (Nacalai Tesque) or 4 to 12% NuPAGE Bis-Tris gels (Thermo Fisher Scientific). The protein concentration was measured using a DC Protein Assay kit (Bio-Rad). After SDS-PAGE, the samples were transferred onto polyvinylidene fluoride membrane (Merck Millipore). The membranes were blocked in T-PBS containing 5% skim milk and incubated with primary antibodies overnight at 4 °C. The primary antibodies used are listed in Table S3. After washing with T-PBS, the membranes were incubated with horseradish peroxidase-conjugated goat anti-mouse IgG or swine anti-rabbit IgG (Dako). Chemiluminescent signals were developed using Clarity Western ECL substrate (Bio-Rad) or Clarity MAX Western ECL substrate (Bio-Rad) and detected using the ImageQuant LAS 4000 imaging system (Cytiva; https:www.cytivalifesciences.com). The signal intensities were quantified using ImageJ (https://imagej.net/ij/) (62).

### *In vitro* ubiquitin binding assay

K48- and K63-linked polyubiquitins were prepared as previously described (63). GST, and GST-tagged yRpn10 and CAPN15 ZF constructs were expressed in *E.coli* BL21 (DE3) by induction with 0.2 mM isopropyl β-D-IPTG for 3 h at 28 °C. Harvested cells were washed with PBS and lysed by sonication in buffer A (50 mM Tris-HCl, pH 7.5, 100 mM NaCl, 10% glycerol). The cell lysates were centrifuged at 20,000×*g* for 15 min, and the supernatants were incubated with glutathione sepharose 4B (Cytiva). After washing three times with buffer A containing 0.1% triton-X100, the GST-tagged proteins immobilized to the sepharose were incubated with 1 μg of K48- or K63-linked polyubiquitin. After washing three times with buffer A containing 0.1% triton-X100, bound polyubiquitins were eluted with one x NuPAGE LDS sample buffer for 10 min at 70 °C.

### Cell culture and treatment, and transfection

HCT116 cells were cultured in Dulbecco’s modified Eagle’s medium (Nacalai Tesque) supplemented with 10% fetal bovine serum (JRH Biosciences) and 1% penicillin-streptomycin (Nacalai Tesque), and maintained in a humidified 37 °C incubator with 5% CO_2_. To inhibit proteasome activity, cells were treated with 10 μM MG132 (Peptide Institute) or 1 μM bortezomib (LC Laboratories) for 4 h prior to harvesting. To inhibit lysosome activity, cells were treated with 100 nM bafilomycin A1 (Adipogen Life Sciences) for 4 h prior to harvesting. To inhibit E1 activity, cells were treated with 1 μM MLN7243 (Active Biochem) for 2 h prior to harvesting. To inhibit cadherin-mediated cell adhesion, cells were treated with 2 mM EDTA. For protein expression, plasmid transfection was performed with PEI-MAX (Polyscience), and the cells were harvested after 24 h. For RNA interference, the On-TARGETplus siRNA SMARTpool (Dharmacon), consisting of four gene-specific siRNAs, was used to knockdown HAKAI, MDM2, FBXW1, and SKP2. For p120-catenin knockdown, two individual On-TARGETplus siRNAs (#1 and #2) (Dharmacon) were used. The siRNA sequences are listed in Table S4. On-TARGET plus Non-targeting pool (Dharmacon) was used as a negative control. Transfection of siRNA was performed using Lipofectamine RNAiMAX transfection reagent (Thermo Fisher Scientific), and after 48 h, the cells were harvested for analysis.

### Cell lysis, immunoprecipitation, and fractionation

Cells were lysed in TNE-N buffer (20 mM Tris-HCl, pH 7.5, 150 mM NaCl, 1 mM EDTA, 1% Nonidet P-40 (NP-40)) containing 25 μM MG132, 10 μM iodoacetamide (Sigma-Aldrich), and protease inhibitor cocktail (Nacalai Tesque). The cell lysates were centrifuged at 20,000*g* for 15 min at 4 °C, and the supernatants were recovered and mixed with SDS-PAGE sample buffer. For immunoprecipitation, the supernatants were incubated with Anti-DDDDK-tag mAb-Magnetic Beads for 1 h at 4 °C or Anti DYKDDDDK tag Antibody Magnetic Beads (Wako) for 4 h at 4 °C. After the beads were washed five times with TNE-N buffer containing the above inhibitors, the bound proteins were eluted with 0.2 mg/ml FLAG peptide (Protein Ark) in TNE-N buffer. Immunoprecipitates were used for downstream analyses. Cell fractionation was performed using a Subcellular Protein Fractionation Kit for Cultured Cells (Thermo Fisher Scientific). Equal proportions of each fraction were used for the western blot analysis.

### *In vitro* deubiquitinase assay

Anti-FLAG immunoprecipitate prepared from CStg cells was incubated with 1 μM USP2 catalytic domain (USP2cc; R&D Systems) for 1 h at 37 °C. The reaction was stopped by the addition of SDS-PAGE sample buffer.

### In-gel digestion and LC-MS analysis

In-gel digestion was performed as previously described (63, 64). Immunoprecipitates were subjected to SDS-PAGE in a short run (1 cm from the top), and visualized using Bio-Safe Coomassie (Bio-Rad). After washing the gels with MilliQ water, the entire protein bands were excised, and diced into small pieces. The gel pieces were de-stained for 1 h in 50 mM ammonium bicarbonate (AMBC) and 30% acetonitrile (ACN), washed for 1 h with 50 mM AMBC/50% ACN, and dehydrated using 100% ACN. Sequence-grade trypsin (Trypsin Gold; Promega, 20 ng/μl in 50 mM AMBC/5% ACN) was added to the dehydrated gel pieces and incubated at 37 °C for 16 h. The peptides were extracted by adding 50% ACN/0.1% TFA, followed by the addition of 70% ACN/0.1% TFA. The extracted peptides were dried by vacuum centrifugation, and resuspended in 0.1% TFA.

Peptides prepared by in-gel digestion were analyzed using an Orbitrap Fusion Lumos Tribrid mass spectrometer connected to an Easy nCL 1200 liquid chromatograph and a nanoelectrospray ion source (Thermo Fisher Scientific) as previously described (64). Peptides were then loaded onto a C18 analytical column (25 cm × 75 μm, 1.6 μm particle size; IonOpticks) and separated using a 150-min gradient of 0 to 80% ACN in 0.1% FA. For data-dependent acquisition of the MS/MS spectra, the most intense ions were selected every 1 s for MS/MS fragmentation by higher-energy collisional dissociation. Data were analyzed using the Sequest HT search program in Proteome Discover 2.4 (Thermo Fisher Scientific). The MS/MS spectra were searched against the SwissProt-reviewed human reference proteome (UniProt). Intensity-based label-free protein quantification was performed using Precursor Ions Quantifier node in Proteome Discoverer 2.4. Peptide identification was filtered at <1% FDR. Results are summarized in Table S2.

### *In vitro* cleavage assay

Ecad-cyt-His, Ub-Ecad-cyt-His, and GST-gp78RING-Ube2g2, were expressed in *E. coli* BL21 (DE3) by induction with 0.4 mM IPTG for 4 h at 30 °C. For Ecad-cyt-His and Ub-Ecad-cyt-His, harvested cells were suspended in buffer A (50 mM Tris-HCl, pH 7.5, 300 mM NaCl, 4 mM imidazole), and lysed using a French Press (Ohtake Works). The cell lysates were centrifuged at 20,000×*g* for 15 min, and the recovered supernatants were applied to Ni^2+^-affinity column (Complete His-tag purification resin; Roche). After washing the column with 10 column volumes of buffer A, purified Ecad-cyt-His and Ub-Ecad-cyt-His were eluted with buffer A containing 150 mM imidazole, and dialyzed against a dialysis buffer (50 mM Tris-HCl, pH7.5, 150 mM NaCl). For GST-gp78RING-Ube2g2, harvested cells were suspended in buffer B (50 mM Tris-HCl, pH 7.5, 150 mM NaCl, 1 mM DTT), and lysed by sonication. The cell lysate was centrifuged at 20,000*g* for 15 min, and the recovered supernatant was incubated with glutathione sepharose 4B. After the beads were washed four times with buffer B and then once with buffer B containing 1 mM EDTA, purified gp78RING-Ube2g2 was obtained by incubating the sepharose beads with prescission protease (Cytiva) in buffer B containing 1 mM EDTA to remove the GST-tag. For preparation of K48(Ub)n-Ecad-cyt-His, reaction was performed with final concentrations of 12 μM Ub-Ecad-cyt-His, 0.5 μM E1 enzyme UBE1 (Life Science), 20 μM gp78RING-Ube2g2, and 400 μM bovine ubiquitin (Sigma-Aldrich) in 20 mM Tris-HCl, pH 7.5, 10 mM MgCl_2_, and 10 mM ATP for 3 h at 37 °C. The reaction mixture was then incubated with His60-Ni-magnetic beads (TaKaRa) in binding buffer (50 mM Tris-HCl, pH 7.5, 150 mM NaCl). After washing the beads four times with the binding buffer, K48(Ub)n-Ecad-cyt-His was eluted with the binding buffer containing 300 mM imidazole, and dialyzed against the dialysis buffer. FLAG-CAPN15 and FLAG-CAPN15CS were purified from WTtg and CStg cells, respectively. After the treatment with 1 μM MLN7243 for 2 h, cells were harvested and lysed in lysis buffer (50 mM Tris-HCl, pH7.5, 150 mM NaCl, 1 mM EDTA, 1% NP-40, 10% glycerol). The cell lysates were centrifuged at 20,000×*g* for 15 min, and the supernatants were incubated with anti-FLAG M2 magnetic beads (Sigma-Aldrich) for 2 h. After washing the beads with the lysis buffer three times, and then twice with elution buffer (50 mM Tris-HCl, pH7.5, 150 mM NaCl, 1 mM EDTA, 10% glycerol), FLAG-CAPN15 and FLAG-CAPN15CS were eluted with 0.2 mg/ml FLAG peptide in the elution buffer. Protein purification was performed at 4 °C. For *in vitro* cleavage assay, FLAG-CAPN15 or FLAG-CAPN15CS (0.25 μg each) was incubated with Ecad-cyt-His or K48(Ub)n-Ecad-cyt-His (1.5 μg each) in reaction buffer (50 mM Tris-HCl, pH 7.5, 150 mM NaCl, 1 mM DTT) containing 10 mM CaCl_2_ or 10 mM EDTA for 1 h at 37 °C. The reactions were stopped by adding SDS-PAGE sample buffer, and then subjected to western blotting.

### *In situ* hybridization and hematoxylin-eosin (HE) staining

C57BL/6N mice (12 weeks old) were euthanized by cervical dislocation to obtain tissue samples. The stomach, colon and skin were removed from C57BL/6N or *Capn15* KO mice, sliced, embedded in Tissue-Tek OCT compounds (Sakura Finetek Japan), and frozen on dry ice. Sections (10 μm thickness) were placed on MAS-coated glass slides (Matsunami Glass). *In situ* hybridization was performed according to the manufacturer’s instructions for nonradioactive *in situ* hybridization (Roche). For *Capn15* sense and antisense probes, digoxigenin (Dig)-labeled riboprobes were synthesized *via in vitro* transcription using a cDNA fragment encoding the mouse CAPN15 ZF region (NM_015830, nt. 231244) subcloned into pBluescript as a template. The riboprobes were hydrolyzed to approximately 150 bases. Hybridization was performed as follows: The sections were fixed with 4% paraformaldehyde (PFA) in PBS. After prehybridization with hybridization buffer (50% formamide, 10% dextran sulfate, 0.6 M NaCl, 10 mM Tris-HCl, pH7.6, 200 μg/ml yeast tRNA, 1 mM DTT) for 2 h at 60 °C, they were hybridized with the probes in hybridization buffer at 60 °C overnight. After washing with 5 × SSC at 60 °C for 5 min, and twice with 0.2 × SSC at 60 °C for 30 min, they were incubated with 1.5% blocking reagent (Roche) in buffer I (100 mM Tris-HCl, pH7.5, 150 mM NaCl), and then with alkaline phosphatase (AP)-conjugated anti-Dig antibody (1:500 dilution) (Roche) in buffer I at room temperature for 1 h. Signal detection was performed by an AP-catalyzing method using a 5-bromo-4-chloro-3-indolyl-phosphate (BCIP)/nitro blue tetrazolium solution (Roche) in reaction buffer (100 mM Tris-HCl, pH9.5, 100 mM NaCl, 50 mM MgCl_2_). For HE staining, the fixed sections were stained with Mayer’s hematoxylin, followed by eosin staining (FUJIFILM Wako Pure Chemicals).

### Immunofluorescence

Cells grown on coverslips (Matsunami Glass) and stomach and colon sections on MAS-coated glass slides were fixed with 4% PFA in PBS for 10 min, and permeabilized with 0.1% triton-X100in PBS for 10 min at room temperature. They were then blocked with 3% bovine serum albumin in PBS, and incubated with anti-E-cadherin (HECD1) mouse monoclonal antibody and anti-β-catenin (D10A8) rabbit monoclonal antibody (for HCT116 and KO cells) or anti-E-cadherin (24E10) rabbit monoclonal antibody (for stomach and colon sections) in 1% bovine serum albumin in PBS overnight at 4 °C. After washing with PBS, the cells and sections were incubated with Alexa Fluor 488-conjugated anti-mouse IgG and Alexa Fluor 594-conjugated anti-rabbit IgG (for HCT116 and KO cells), or Alexa Fluor 488-conjugated anti-rabbit IgG (for stomach and colon sections) (Thermo Fisher Scientific). After washing with PBS, they were incubated with 4′,6-diamidino-2-phenylindole (DAPI; Sigma-Aldrich) in PBS for 5 min at room temperature, mounted using Prolong Diamond Antifade Mountant (Thermo Fisher Scientific), and observed by LSM710 laser-scanning confocal microscope with a Plan-Apochromat 63 × 1.4 oil objective lens (Carl Zeiss). Image analysis was performed using ZEN 2012 imaging software (Carl Zeiss) and ImageJ.

### Cell adhesion assay

Cell adhesion was assessed using E-cadherin-Fc-coated 96-well plates (SOMAR Corp.) according to the manufacturer’s instructions with slight modifications. HCT116 and KO cells were detached using accutase (Nakalai Tesque), suspended in Hank’s balanced salt solution with Ca^2+^ and Mg^2+^, and seeded at a density of 3 × 10^4^ cells/well. The plates were incubated for 15 min at 37 °C to allow cell adhesion, and non-adherent cells were removed by gentle washing with Hank’s balanced salt solution with Ca^2+^ and Mg^2+^. Adherent cells were fixed and stained with 0.4% crystal violet in methanol for 30 min, followed by extensive washing with water. After air-drying, the dye was solubilized in 33% acetic acid, and absorbance at 595 nm was measured using a microplate reader (Bio-Rad). Adhesion was quantified as the value normalized to that of HCT116 cells.

### Protein preparation from mouse tissues

Mucosae were scraped from the stomach and colon of WT and *Capn15* KO littermates (18 or 26 weeks old), and the back skin was excised from WT and KO littermates (postnatal day 2). They were then homogenized in homogenizing buffer (10 mM Tris-HCl, pH7.5, 1 mM EDTA, 1 mM DTT) containing a protease inhibitor cocktail using a micro homogenizer (Microtec). After centrifugation at 20,000×*g* for 15 min at 4 °C, the supernatants were used for western blotting.

### qRT-PCR

Total RNA was isolated using TRIzol Reagent (Thermo Fisher Scientific). First-strand cDNA was synthesized using the PrimeScript RT Master Mix (Perfect Real Time) (TaKaRa). PCR was performed on a LightCycler 480 System II (Roche) using TB Green Premix Ex Taq II (Tli RNaseH Plus) (TaKaRa). The primer sets used are as follows: 5′-agcctctggatagagaagcca-3′ and 5′-ttcatcacggaggttcctgg-3′ for mouse *Cdh1* mRNA (E-cadherin mRNA), and 5′-cctcgtcccgtagacaaaatg-3′ and 5′-tctccactttgccactgcaa-3′ for mouse *Gapdh* mRNA.

## Data availability

Mass spectrometry proteomics data were deposited in the ProteomeXchange Consortium *via* the jPOSTrepo with the dataset identifier PXD059028.

## Supporting information

This article contains supporting information.

## Conflict of interest

The authors declare that they have no conflicts of interest with the contents of this article.
