# Supplementary material for: CAPN15 is a non-proteasomal, ubiquitin-directed calpain protease that regulates cell adhesion by cleaving E-cadherin
Source: J Biol Chem. 2025 Dec 9;302(1):111034. doi: 10.1016/j.jbc.2025.111034 (PMC12805371; doi:10.1016/j.jbc.2025.111034)
Supplement: Supporting Information [file mmc1.pdf]

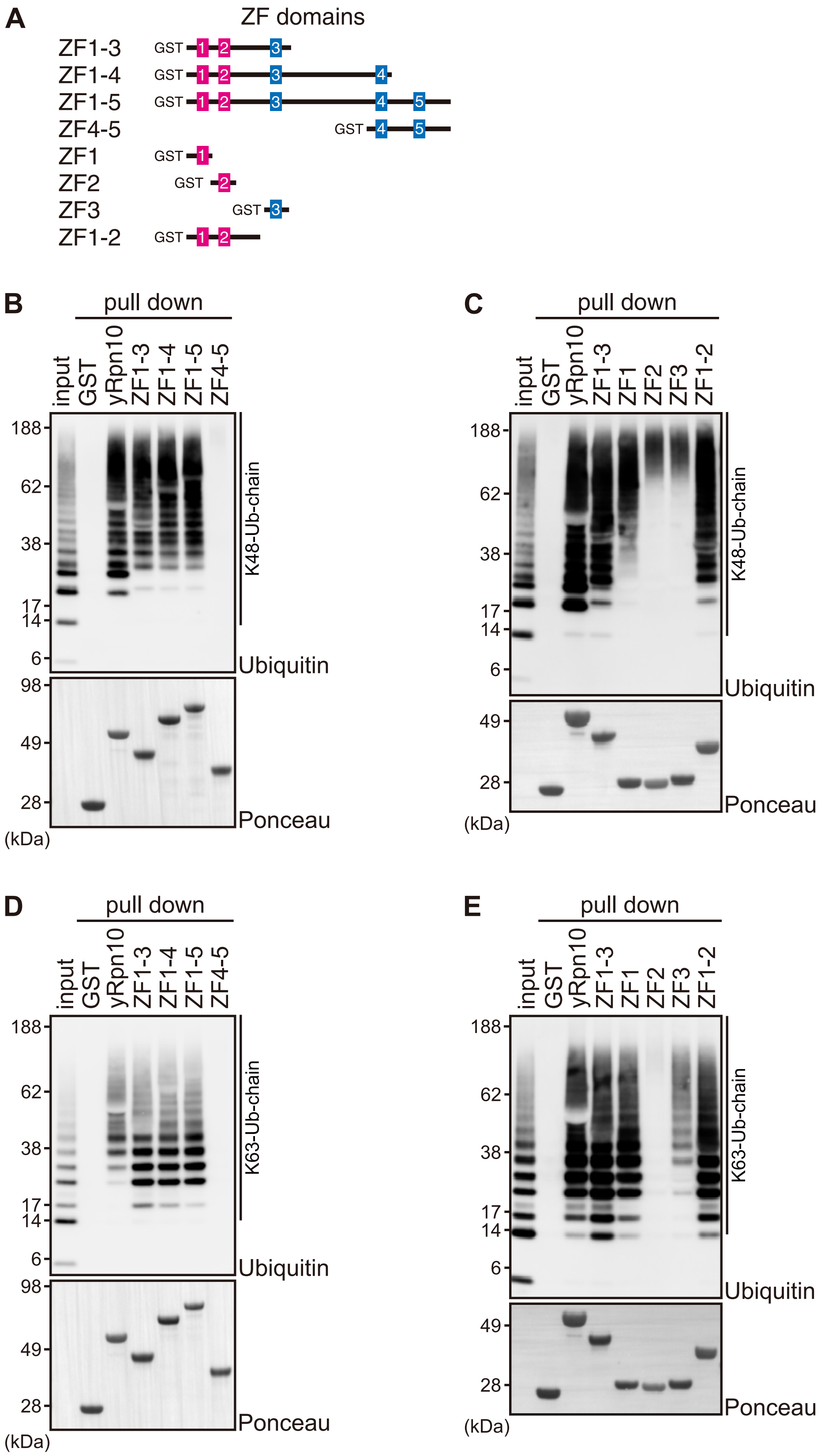

Figure S1. *In vitro* ubiquitin binding assay of the CAPN15 ZF domains.

(A) Schematic of GST-tagged ZF constructs. (B and C) K48- or (D and E) K63-linked polyubiquitin chains were pulled down with GST or GST-tagged ZF constructs immobilized on glutathione sepharose beads. GST-yRpn10 (ubiquitin-binding subunit of budding yeast proteasome) was used as a positive control. The bound polyubiquitins were detected by western blotting using anti-ubiquitin antibodies (upper panels). GST-tagged proteins were stained by Ponceau S (lower panels).

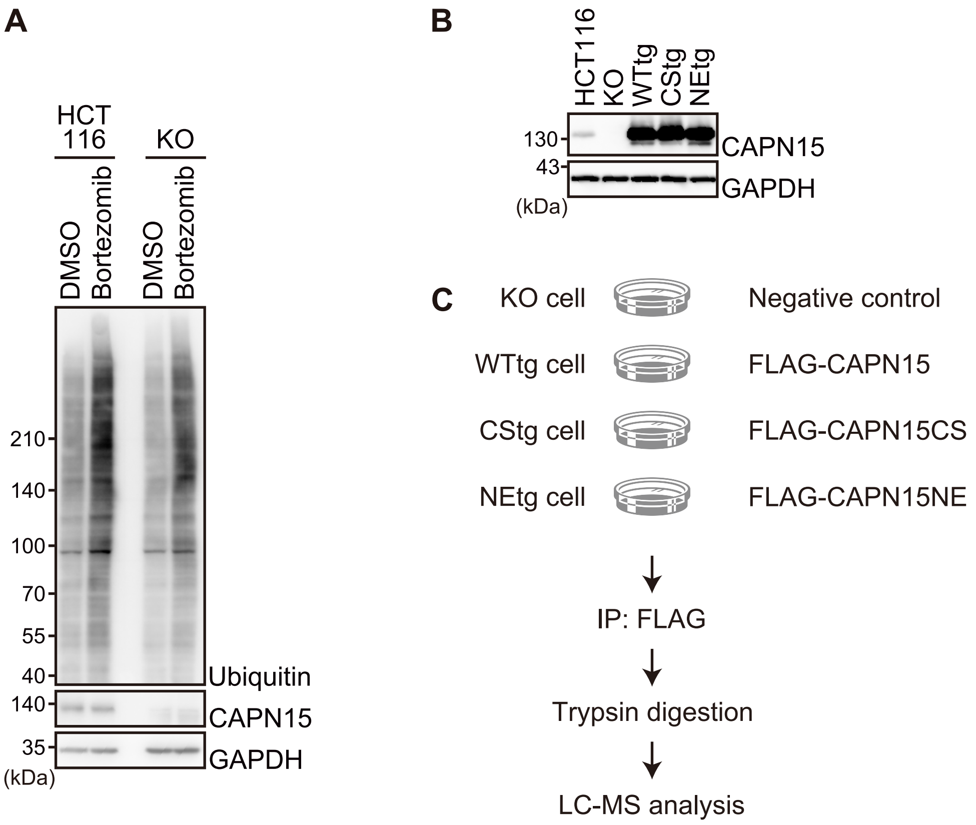

Figure S2. Proteomic analysis of CAPN15-interacting proteins.

(A) Western blot analysis of ubiquitinated proteins in HCT116 and KO cells treated with DMSO (vehicle) or 1 μM bortezomib. (B) Expression levels of FLAG-CAPN15, FLAG-CAPN15CS, and FLAG-CAPN15NE in WTtg, CStg, and NEtg cells, respectively, were analyzed by western blotting, compared to that of endogenous CAPN15 in HCT116 cells. (C) Experimental procedures for the proteomic analysis of CAPN15-interacting proteins (see also Experimental Procedures section).

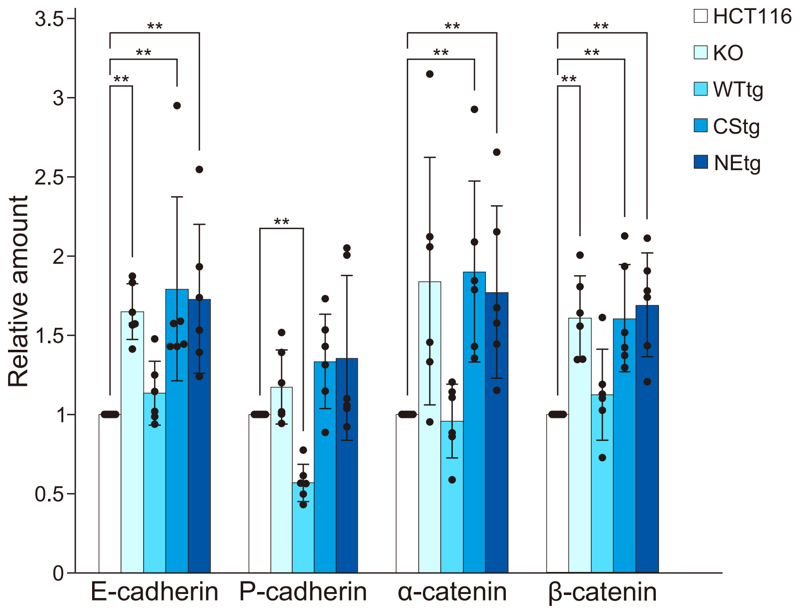

Figure S3. Quantification of E- and P-cadherins and α- and β-catenins in HCT116, KO, WTtg, CStg, and NEtg cells. Band intensities in Fig. 4A are quantified and normalized by that of GAPDH. Relative intensity of each protein in HCT116 cells was defined as 1. The graph represents the mean ± SD with individual data points (n=6). $*P < 0.05$, $**P < 0.01$ by Steel-Dwass test.

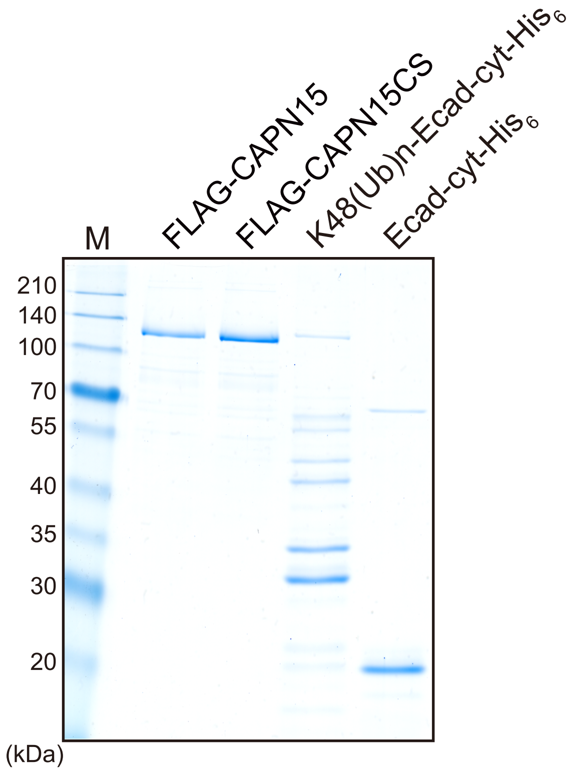

Figure S4. Purification of recombinant CAPN15 and E-cadherin substrates.

Purification of the proteins used in the *in vitro* cleavage assay in Fig. 5. Purified proteins were visualized by Coomassie staining.

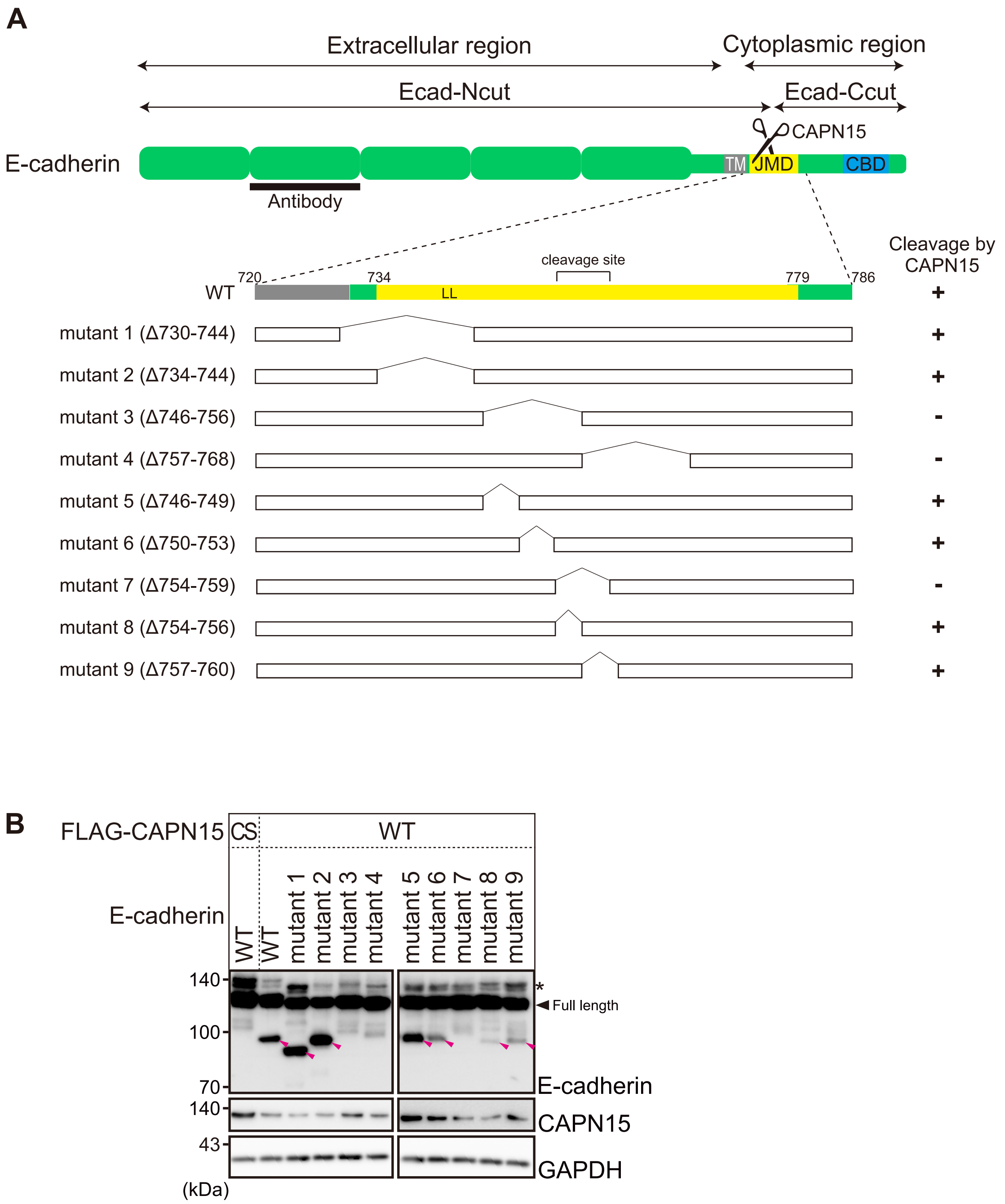

Figure S5. Determination of the E-cadherin cleavage site.

(A) Schematic of the domain organization of E-cadherin and its deletion mutants. The domains are shown in different colors with the amino acid residue numbers. A summary of cleavage by CAPN15 is shown on the right. TM, transmembrane domain; JMD, juxtamembrane domain; CBD, catenin-binding domain; LL, dileucine motif. (B) E-cadherin constructs were transfected into KO cells with FLAG-CAPN15 or FLAG-CAPN15CS as indicated combinations. The cell lysates were analyzed by western blotting. Red arrowheads indicate proteolytic fragments. The asterisk indicates a non-specific band.

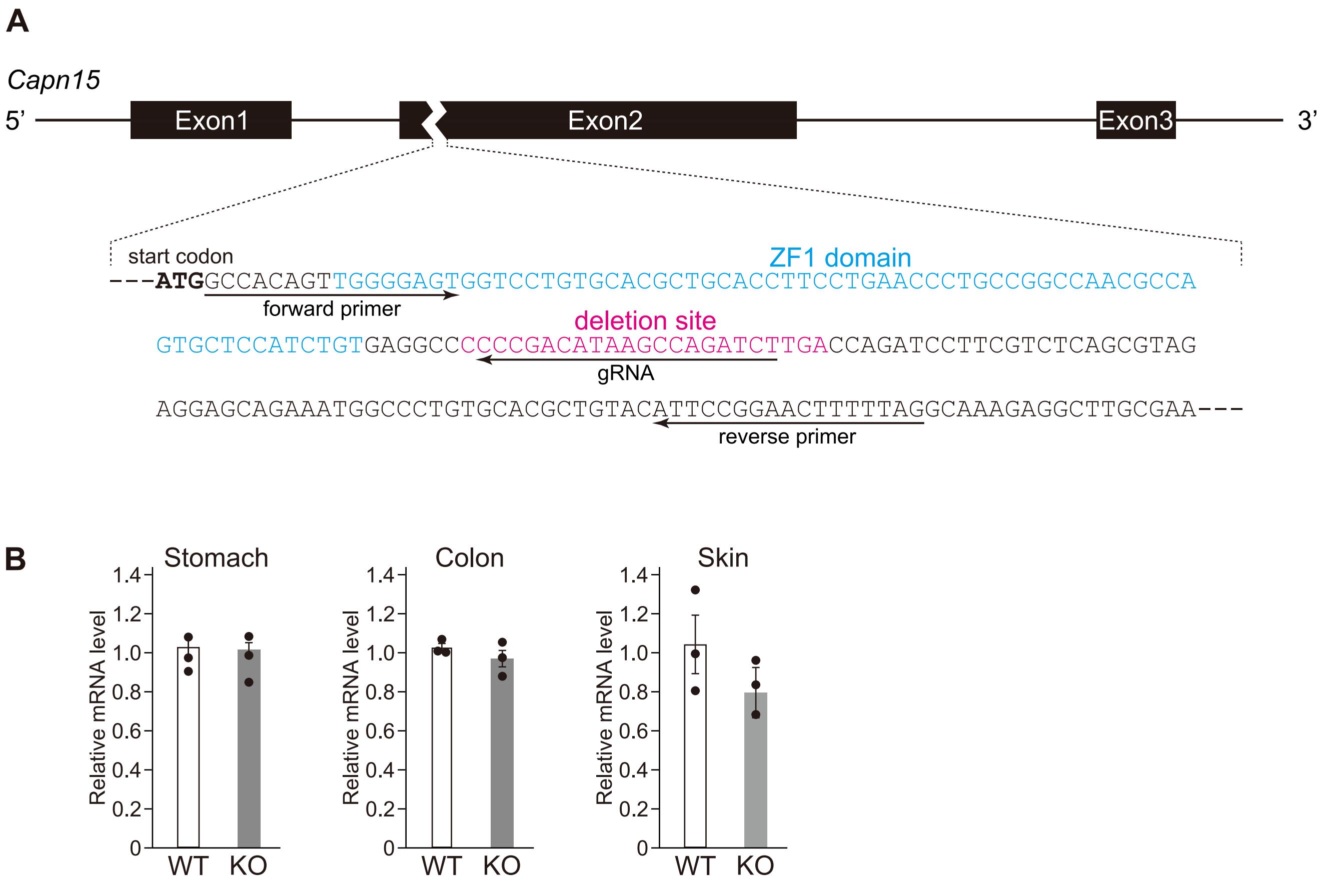

Figure S6. Generation of *Capn15* KO mice using CRISPR-Cas9 system.

(A) Schematic of the Cas9-gRNA-targeted site in the mouse *Capn15* genomic locus. The sequences of the start codon, the ZF1 domain, and the deletion site are shown in bold, blue, and magenta, respectively. The gRNA sequence and primers used for PCR genotyping are also shown. The resulting KO allele has a 23-bp deletion starting 81-bp downstream of the start codon. (B) The mRNA levels of E-cadherin were quantified by qPCR in the stomach, colon, and skin of the same wild-type (WT) and *Capn15* KO (KO) littermates as those used in Fig. 7C. The graph represents the mean ± SD with individual data points (n=3). No significant difference was detected by Welch's t-test.

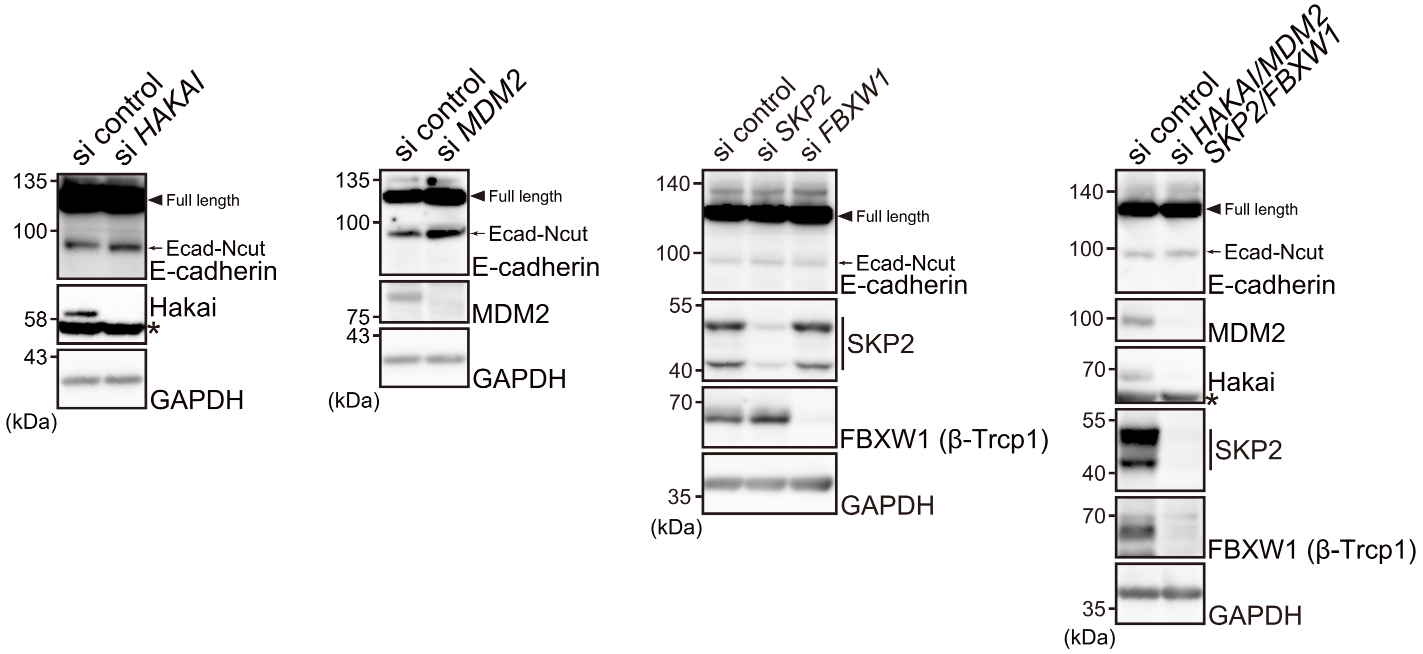

Figure S7. Search for knockdown of E3 ubiquitin ligases on E-cadherin cleavage by CAPN15.

HCT116 cells were transfected with indicated siRNAs. Forty-eight hours after the transfection, cells were treated with bafilomycin A1 for 4 h. The cell lysates were subjected to western blot analysis. Neither individual nor combined knockdown of four E3 ubiquitin ligases that have been reported to act on E-cadherin or β-catenin suppressed E-cadherin cleavage by CAPN15, indicating the involvement of other E3 ligases responsible for this process. The asterisks indicate non-specific bands.

Table S1. Abbreviations of CAPN15 and E-cadherin constructs, and derived cell lines used in this study

Abbreviation	Full name	Description
CS	Protease-inactive CAPN15 C552S mutant	CAPN15 carrying a point mutation (C552S) that abolishes protease activity
NE	CAPN15 NE mutant	CAPN15 mutant in which each TF motif and the corresponding TL dipeptide in the five ZF domains are replaced with NE
WTtg	FLAG-CAPN15 WT transgenic	KO cell stably expressing FLAG-CAPN15 WT
CStg	FLAG-CAPN15 CS transgenic	KO cell stably expressing FLAG-CAPN15 CS
NEtg	FLAG-CAPN15 NE transgenic	KO cell stably expressing FLAG-CAPN15 NE
Ecad-cyt-His	E-cadherin cytoplasmic region-His	E-cadherin cytoplasmic region with C-terminal His-tag
Ub-Ecad-cyt-His	Ubiquitin-E-cadherin cytoplasmic region-His	E-cadherin cytoplasmic region with N-terminal monoubiquitin-tag and C-terminal His-tag
K48(Ub)n-Ecad-cyt-His	K48-linked polyubiquitin-E-cadherin cytoplasmic region-His	E-cadherin cytoplasmic region with N-terminal K48-linked polyubiquitin-tag and C-terminal His-tag

Table S3. Antibodies used in this study

	RRID	Supplier	Catalog No.	Dilution used WB	IF
CAPN15 (D-5)	AB_3099640	Santa Cruz Biotechnology	sc-514406	1:1000	
CBLL1/HAKAI	AB_10732814	Proteintech	21179-1-AP	1:1000	
E-cadherin (HECD-1)	AB_3099641	Takara	M106	1:1000 - 1:2000	1:100
E-cadherin	AB_10697811	Proteintech	20874-1-AP	1:1000	
E-cadherin (24E10)	AB_2291471	Cell Signaling Technology	#3195	1:1000	1:100
p120-catenin (6H11)	AB_2086394	Santa Cruz Biotechnology	sc-23873	1:1000	
β-TrCP (D13F10)	AB_10545763	Cell Signaling Technology	#4394	1:1000	
FLAG (M2)	AB_259529	Sigma-Aldrich	F3165	1:2000	
GAPDH (6C5)	AB_627679	Santa Cruz Biotechnology	sc-32233	1:5000	
GAPDH (3H12)	AB_590502	MBL	M171-3	1:2000	
His (OGHis)	AB_1059773	MBL	D291-3	1:3000	
Histone H3	AB_331563	Cell Signaling Technology	#9715	1:1000	
MDM2 (SMP14)	AB_627920	Santa Cruz Biotechnology	sc-965	1:250	
MDM2 (D1V2Z)	AB_2784534	Cell Signaling Technology	#86934	1:1000	
Na^+/K^+ ATPase (EP1845Y)	AB_1310695	Abcam	ab76020	1:10000	
P-cadherin (1G10F7)	AB_2881678	Proteintech	66295-1-Ig	1:500	
Skp2	AB_2187647	Proteintech	15010-1-AP	1:1000	
Ubiquitin (A5, HRP-conjugated)	AB_2241297	Santa Cruz Biotechnology	sc-166553	1:500	
Ubiquitin (P4D1, HRP-conjugated)	AB_628423	Santa Cruz Biotechnology	sc-8017	1:500	
Ubiquitin (FK2)	AB_592937	MBL	D058-3	1:1000	
αE-catenin (2B7B8)	AB_2881612	Proteintech	66221-1-Ig	1:500	
β-catenin (D10A8)	AB_11127855	Cell Signaling Technology	#8480	1:1000	1:100
HRP-conjugated goat anti-mouse IgG	AB_2617137	Dako	P0447	1:2000 - 1:3000	
HRP-conjugated swine anti-rabbit IgG	AB_2617141	Dako	P0399	1:2000 - 1:3000	
Alexa Fluor 488-conjugated goat anti-mouse IgG	AB_2534088	Thermo Fisher Scientific	A-11029		1:1000
Alexa Fluor 488-conjugated goat anti-rabbit IgG	AB_2576217	Thermo Fisher Scientific	A-11034		1:500 - 1:1000
Alexa Fluor 594-conjugated goat anti-rabbit IgG	AB_2534079	Thermo Fisher Scientific	A-11012		1:500

Table S4. siRNAs used in this study

Target gene	siRNA sequences (5' to 3')
CBLL/HAKAI	#1; GACAAGAUAUAGACCGUAU
	#2; GGGAAUGAGUCCUGGUAUA
	#3; GGGGUGAGCUGUUUGCAAA
	#4; AUCAACCAUCGCCAUAUG
MDM2	#1; GCCAGUAUAUUAUGACUAA
	#2; GAACAAGAGACCCUGGUUA
	#3; GAAUUUAGACAACCUGAAA
	#4; GAUGAGAAGCAACAACAACAUA
FBXW1	#1; UGACAACACUAUCAGAUUA
	#2; CACAUAAACUCGUAUCUUA
	#3; GACCUUAAAUGGACACAAA
	#4; ACACCGAGCUGCUGUCAAU
SKP2	#1; UGUCAAUACUCUCGCAAAA
	#2; UCGGUGCUAUGAUAUAUAAUA
	#3; GGUAUCGCCUAGCGUCUGA
	#4; GGAUGUGACUGGUCGGUUG
CTNND1/p120-catenin	#1; GGAAUGUGAUGGUUUAGUU
	#2; UAGCUGACCUCCUGACUAA
